# Severe asthma features in children: a case–control online survey

**DOI:** 10.1186/s13052-016-0217-z

**Published:** 2016-01-22

**Authors:** Silvia Montella, Eugenio Baraldi, Salvatore Cazzato, Raffaele Aralla, Mariangela Berardi, Luigia Maria Brunetti, Fabio Cardinale, Renato Cutrera, Fernando Maria de Benedictis, Emanuela di Palmo, Sabrina Di Pillo, Grazia Fenu, Stefania La Grutta, Enrico Lombardi, Giorgio Piacentini, Francesca Santamaria, Nicola Ullmann, Franca Rusconi, the Italian Pediatric Severe Asthma Network (IPSAN) on behalf of the Italian Society of Pediatric Respiratory Diseases (SIMRI)

**Affiliations:** Department of Translational Medical Sciences, Federico II University, Naples, Italy; Women’s and Children’s Health Department, University of Padua, Padua, Italy; Department of Pediatrics, S. Orsola-Malpighi Hospital, University of Bologna, Bologna, Italy; High Altitude Pediatric Asthma Center-Pio XII Institute, Misurina, Italy; Department of Pediatrics, University of Bari “Aldo Moro”, Bari, Italy; Department of Pediatrics, Pediatric Hospital “Giovanni XXIII”, University of Bari, Bari, Italy; Respiratory Unit, Department of Pediatric Medicine, Bambino Gesù Children Hospital, Rome, Italy; Department of Mother and Child Health, Salesi Children’s Hospital, Ancona, Italy; Department of Pediatrics, University of Chieti, Chieti, Italy; Pediatric Pulmonary Unit, A. Meyer Children’s University Hospital, Florence, Italy; Institute of Biomedicine and Molecular Immunology of National Research Council, University of Palermo, Palermo, Italy; Department of Science for Health Promotion and Mother and Child, University of Palermo, Palermo, Italy; Pediatric Section, Department of Life and Reproduction Sciences, University of Verona, Verona, Italy; Epidemiology Unit, A. Meyer Children’s University Hospital, Viale Pieraccini 24, 50139 Florence, Italy

**Keywords:** Asthma, Atopy, Children, Lung function, Quality of life

## Abstract

**Background:**

Very few studies have explored the distinguishing features of severe asthma in childhood in Europe, and only one study was conducted in Southern Europe. The aim of this study was to provide a detailed characterization of children with severe asthma treated in specialized pediatric asthma centers across Italy.

**Methods:**

We conducted a web-based data collection of family, environmental, clinical and laboratory characteristics of 41 patients aged 6–17 years with severe asthma, defined according to the recent guidelines of the European Respiratory Society and the American Thoracic Society, and 78 age-matched peers with non-severe persistent asthma. The patients have been enrolled from 16 hospital-based pediatric pulmonology and allergy centers in Northern, Central, and Southern Italy. Logistic regression analysis assessed the relationship between patients’ characteristics and severe asthma or non-severe persistent asthma.

**Results:**

Features independently and significantly associated with severe asthma included lifetime sensitization to food allergens [Odds ratio (OR), 4.73; 95 % Confidence Interval (CI), 1.21–18.53; *p* = 0.03], lifetime hospitalization for asthma (OR, 3.71; 95 % CI, 1.11–12.33; *p* = 0.03), emergency-department visits for asthma during the past year (OR = 11.98; 95 % CI, 2.70–53.11; *p* = 0.001), and symptoms triggered by physical activity (OR = 12.78; 95 % CI, 2.66–61.40; *p* = 0.001). Quality-of-life score was worse in patients with severe asthma than in subjects with non-severe persistent asthma (5.9 versus 6.6, *p* = 0.005). Self-perception of wellbeing was compromised in more than 40 % of patients in both groups. Children with severe asthma had lower spirometric z scores than non-severe asthmatic peers (all *p* < 0.001), although 56 % of them had a normal forced expiratory volume in 1 s. No differences were found between the two groups for parental education, home environment, patients’ comorbidities, adherence to therapy, exhaled nitric oxide values, and serum eosinophils and IgE .

**Conclusions:**

As expected, children with severe asthma had more severe clinical course and worse lung function than peers with non-severe persistent asthma. Unlike previous reports, we found greater sensitization to food allergens and similar environmental and personal characteristics in patients with severe asthma compared to those with non-severe persistent asthma. Psychological aspects are compromised in a large number of cases and deserve further investigation.

**Electronic supplementary material:**

The online version of this article (doi:10.1186/s13052-016-0217-z) contains supplementary material, which is available to authorized users.

## Background

The vast majority of asthmatic children exhibit a mild or moderate form of the disease, and only a small proportion requires aggressive maintenance therapy for several months or remains difficult-to-treat [1]. Nevertheless, these children account for a relatively large amount of resource expenditure among asthmatic patients [1, 2]. In the last few years, guidelines and consensus documents have been implemented to address the problem of poorly controlled asthma and to provide a uniform definition of severe asthma (SA) in children [3–5]. A Global Allergy and Asthma European Network (GA2LEN) task force suggested a reasoned approach to children with problematic SA and proposed recommendations for its assessment and treatment [5]. Recently, a joint Task Force supported by the European Respiratory Society (ERS) and the American Thoracic Society (ATS) reviewed the terminology and provided evidence-based recommendations for defining and treating SA in children and adults [2].

So far, very few studies have explored the distinguishing features of SA in childhood [6–10], and due to the lack of a recognized international definition of SA, inclusion criteria and study design were heterogeneous. Two studies were carried out in the United States [6, 7]. The first was a large study on patients with either severe or difficult-to-treat asthma enrolled in the TENOR (The Epidemiology and Natural History of Asthma: Outcomes and Treatment Regimens) study [6]. The other study recruited children across five large academic centers participating in the Severe Asthma Research Program supported by the National Heart, Lung and Blood Institute [7]. Only three studies were performed in Europe, specifically in France [8], Sweden [9], and Norway [10], and enrolled smaller sample sizes than those from the United States. Furthermore, while studies from Northern Europe recruited children in several hospitals and university clinics [9, 10], the French study enrolled patients from a single specialized center [8]. Therefore, data on pediatric SA in Europe, and particularly in Southern Europe, are still scarce. Identifying the distinctive characteristics of children with SA may be useful not only for improving scientific knowledge of SA-related risk factors and phenotypes, but also as the basis for developing tailored approaches to asthma-patient management.

The aim of this study was to provide a detailed characterization of children treated in specialized pediatric asthma centers across Italy who complied with the recent ERS/ATS definition of SA [2], and to compare these children with age-matched peers with non-severe persistent asthma (NSPA).

## Methods

### Patients

In the current multicenter, case–control study, children and adolescents aged 6–17 years with SA and NSPA were enrolled at outpatient hospital clinics by trained pediatric pulmonologists and allergists between May 2013 and June 2014. The study was promoted and supported by the Italian Society of Pediatric Respiratory Diseases (SIMRI). Inclusion criteria for all subjects were: confirmed diagnosis of asthma, defined as ≥12 % change in FEV_1_ after bronchodilator administration; evaluation and management of the patient at the study center for more than three months in order to exclude differential diagnoses (Additional file 1) and address comorbidities and contributory factors. Patients were defined as having SA if they required treatment with high doses of inhaled corticosteroids (Additional file 2) *plus* at least another controller for at least 6 months in the previous year and still ongoing at the time of recruitment. They also had to meet at least one of the following criteria in the preceding year: at least two asthma exacerbations requiring systemic corticosteroids for more than 3 days; daytime and/or nighttime asthmatic symptoms and/or daily activity limitation more than twice a week for at least 3 months; persistent airflow obstruction despite administration of oral steroids and bronchodilators for at least 2 weeks (Additional file 2).

For each SA case enrolled, the centers had to recruit 2 age-matched (+/−2 years at most) peers with NSPA (occasional asthmatic symptoms and less than 2 exacerbations requiring systemic steroids in the preceding year) controlled by lower doses of inhaled corticosteroids than those required for inclusion in the SA group (Additional file 2) [1]. The study was approved by the ethics committee of the coordinating center (“Anna Meyer” Pediatric University Hospital, Florence; approval number: 245, year 2012), and informed written consent was obtained from the parent/legal guardian of each child prior to the study inclusion. All children agreed to take part in the study.

### Clinical and laboratory assessment

Parents of all the enrolled subjects were interviewed by means of a modified version of the SIDRIA questionnaire [11], including enquiries regarding the following items: parents’ country of birth, education, history of asthma and other allergic or respiratory diseases, smoking habits, and working days lost in the past year because of their child’s asthma; indoor and outdoor environment, including pets, dampness in the home, number of cohabitants, and heavy traffic in the street of residence; the patient’s physical activity, comorbidities, and history of anaphylaxis, atopic eczema, allergic rhinitis, and food allergies (diagnosed on the basis of history of anaphylactic reactions or positive food challenge). Additional enquiries concerning the twelve months preceding the study included: asthma symptoms and their triggering factors; asthma medications; adherence to therapy; use of healthcare services. Information about physical activity and active smoking was obtained directly from the child if aged 12 years or over. Lifetime sensitization assessed by skin-prick testing (defined positive if at least 1 allergen had a wheal diameter ≥3 mm larger than the negative control) and/or serum specific IgEs were obtained from the patients’ clinical charts. All children were assessed for self-perception of health-related quality of life (HR-QoL) using the Italian version of the Paediatric Asthma Quality of Life Questionnaire [12, 13]. Children aged 12 years or over were also assessed for self-perception of wellbeing using the Italian version of the WHO-5 Wellbeing Index [14], available on https://www.psykiatri-regionh.dk/who-5/who-5-questionnaires/Pages/default.aspx. A score below 13 indicated poor wellbeing.

At the study entry, body mass index (BMI) was calculated as weight (kg)/height (m) squared. Obesity was defined as BMI >95^th^ percentile [15]. Each investigator scored the patient’s ability to use the inhaler properly on a scale ranging from 0 (totally incorrect execution) to 10 (perfect execution). Blood eosinophil count and total serum IgE levels were determined. The fraction of exhaled nitric oxide (FE_NO_) was measured before spirometry at an exhalation flow rate of 50 mL/s, according to international guidelines [16]. Short-acting and/or long-acting bronchodilators and/or leukotriene antagonists were respectively withheld 8, 24, and 72 h before spirometry. Spirometry was performed in accordance with international recommendations [17], and z scores were calculated for FVC, FEV_1_, FEV_1_/FVC ratio, and FEF_25–75_ [18]. A FEV_1_ z score ≥ −1.64 was considered normal.

### Data retrieval and monitoring

For data collection, a centralized Web-based system was developed by the CINECA Inter University Consortium (Bologna, Italy) based on secure AXMR® technology. Registered centers accessed the database directly online using a personal identification and password. The system automatically performed eligibility checks and then confirmed or refused the patient’s enrolment. Data were entered by centers on online electronic forms and stored at the quality and security procedure-certified CINECA Data Center. The web-reporting system was always available to analyze data, with information updated daily. Data managers (S.M. and F.R.) were properly trained to use the web-data management system.

### Statistical analysis

Results are expressed as medians and ranges for continuous variables, and as percentages for categorical data. Comparisons were made using Fisher’s exact test and the Mann–Whitney *U* test. Characteristics demonstrating significant differences between SA and NSPA in the univariate analysis were further analyzed by binary logistic regression, adjusting for age, gender, center, and mutual relationships. We did not include in the multivariable analysis variables which were highly correlated such as age at anti-asthma maintenance treatment start, hospital admissions for asthma during the past year, oral steroids for asthma exacerbations, and parents with workdays lost; we included instead parental smoking, which was of borderline significance at the univariate analysis. A two-sided *p* < 0.05 was considered significant. Data were analyzed with SPSS-PC 13.0 (SPSS Inc., Chicago, IL).

## Results

Forty-one children with SA and 78 with NSPA were enrolled from 16 centers. Subjects with SA had a higher prevalence of lifetime sensitization to food allergens than their NSPA peers (*p* = 0.04), while no significant difference for sensitization to aeroallergens was observed between the two groups (Table 1). Children with SA started anti-asthma maintenance treatment significantly earlier than NSPA peers (*p* = 0.04). Moreover, during the preceding 12 months, SA subjects had more frequent episodes of hospitalization and emergency-department visits for asthma, oral steroids for asthma exacerbations, symptoms triggered by physical activity (all *p* values < 0.001), and nocturnal symptoms (*p* = 0.049). None of the patients from either group had been admitted to intensive care units during the previous year. Comorbidities (i.e. rhinoconjunctivitis, obesity, and symptoms of chronic sinusitis and of gastroesophageal reflux) were equally distributed between the two groups, and the degree of physical activity was similar. No difference in physical activity was found between boys and girls in both groups. Only one patient was an active smoker.

**Table 1:** Personal characteristics of children with severe asthma (SA) and non-severe persistent asthma (NSPA)

	SA	NSPA	*p*
	(*n* = 41)	(*n* = 78)	
Personal characteristics			
Age at the study, yrs	12 (6–17)	12 (6–17)	0.8
Male gender	27 (66)	47 (60)	0.7
Lifetime atopic sensitization to			
house dust mites	31 (76)	59 (76)	1
pets dander	21 (51)	47 (60)	0.4
moulds	13 (32)	24 (31)	1
pollen	28 (68)	61 (78)	0.3
cow milk proteins/egg/peanuts	15 (37)	14 (18)	0.04
History of anaphylactic reactions	6 (15)	10 (13)	0.8
Current atopic eczema	10 (24)	11 (14)	0.2
Current food allergies	6 (15)	9 (11)	0.8
Age at onset of asthmatic symptoms, yrs	3 (0–11)	2 (0–12)	0.8
Age at physician’s diagnosis of asthma, yrs	5 (0–11)	6 (0–13)	0.06
Age at anti-asthma maintenance treatment start, yrs	6 (1–12)	7 (1–13)	0.04
Lifetime hospital admissions for asthma	33 (80)	30 (38)	<0.001
Hospital admissions for asthma during the past year	12 (29)	3 (4)	<0.001
Emergency-department visits for asthma during the past year	19 (46)	8 (10)	<0.001
Oral steroids for asthma exacerbation in the past year	32 (78)	34 (44)	<0.001
Nocturnal symptoms between exacerbations	22 (54)	26 (33)	0.049
Asthmatic symptoms triggered by			
physical activity	36 (88)	41 (53)	<0.001
allergens	24 (59)	44 (56)	0.8
airway infections	28 (68)	41 (53)	0.1
fog, humid or cold air, or perceivable odors	19 (46)	29 (37)	0.4
tobacco smoke	9 (22)	10 (13)	0.2
Comorbidities			
Current rhinoconjunctivitis	23 (56)	48 (61)	0.7
Obesity	4 (10)	7 (9)	1
Symptoms of chronic sinusitis	4 (10)	2 (3)	0.2
Symptoms of gastro-esophageal reflux	6 (15)	7 (9)	0.4
Regularly playing a sport	26 (63)	51 (65)	0.8
Physically active >5 h/wk	10 (24)	21 (27)	0.8

Data are presented as number of patients (%) or median values (range)

At recruitment, additional controllers to inhaled corticosteroids used by the study subjects were long-acting inhaled bronchodilators (95 % of children with SA *versus* 47 % of patients with NSPA, *p* < 0.001), oral Montelukast (68 % *versus* 36 %, *p* < 0.001), maintenance oral steroids (2 % *versus* 0 %, *p* = 0.3), oral sustained release methylxanthines (7 % *versus* 0 %, *p* = 0.04), or subcutaneous Omalizumab (32 % *versus* 0 %, *p* < 0.001). Twelve percent of patients with SA and 6 % of children with NSPA had missed at least one dose of controller medications more than once a week during the previous six months (*p* = 0.3). The inhaler technique was good for both groups, with a median score of 9 (range, 6–10) for SA and 9 (range, 5–10) for NSPA subjects (*p* = 0.6).

Parents of children with SA had lost more working days in the previous year (12 versus 5, *p =* 0.01). No significant differences were observed between the groups for other parental and environmental characteristics, although children with SA tended to have a higher prevalence of smoking parents (54 % *versus* 36 %, *p* = 0.08) (Table 2).

**Table 2:** Family and environmental characteristics of children with severe asthma (SA) and non-severe persistent asthma (NSPA)

	SA	NSPA	*p*
	(*n* = 41)	(*n* = 78)	
Family characteristics			
At least one parent born in Italy	37 (90)	73 (94)	0.5
Parental education beyond high school	6 (15)	19 (24)	0.2
At least one asthmatic parent	36 (88)	71 (91)	0.7
Parents with workdays lost during the past year	16 (39)	16 (21)	0.049
Environmental characteristics			
At least one smoking parent	22 (54)	28 (36)	0.08
Number of cohabitants	3 (1–7)	3 (1–6)	1
Dampness in the home	17 (41)	27 (35)	0.5
Pets at home	10 (24)	23 (29)	0.7
Heavy traffic in the residence street	8 (19)	13 (17)	0.8

Data are presented as number of patients (%) or median values (range)

The multiple logistic regression analysis showed that lifetime sensitization to food allergens, lifetime hospital admission for asthma, emergency-department visits during the past year, and symptoms triggered by physical activity were independently associated with SA (Table 3).

**Table 3:** Characteristics associated with severe asthma at logistic regression analysis in 41 patients with SA compared to 78 NSPA children

	OR	95 % CI	*p*
Lifetime atopic sensitization to cow’s milk proteins/egg/peanuts	4.73	1.21–18.53	0.03
Lifetime hospital admission for asthma	3.71	1.11–12.33	0.03
Emergency-department visit for asthma during the past year	11.98	2.70–53.11	0.001
Nocturnal symptoms between exacerbations	1.16	0.34–3.97	0.8
Asthmatic symptoms triggered by physical activity	12.78	2.66–61.40	0.001
At least one smoking parent	1.54	0.45–5.25	0.5

*OR* odds ratio; *CI* confidence interval

Adjustments were made for all the listed factors and for age, gender, and center

HR-QoL was significantly worse in SA than NSPA cases, while the WHO-5 Wellbeing Index was similar in both groups, with 12 SA (44 %) and 20 NSPA children (43 %) having an index value lower than 13 (Table 4). Compared with girls, boys showed lower HR-QoL scores (physical activity limitation domain: 5.2 *versus* 6.5, *p* = 0.03; symptom domain: 5.5 *versus* 6.3, *p* = 0.02; and total score: 5.8 *versus* 6.5, *p* = 0.02), but a similar WHO-5 Wellbeing Index (58 % *versus* 44 %, *p* = 0.4).

**Table 4:** Quality-of-life and wellbeing scores in children with severe asthma (SA) and non-severe persistent asthma (NSPA)

	SA	NSPA	*p*
	(*n* = 41)	(*n* = 78)	
HR-QoL scores			
Physical activity limitation domain	5.8 (2.0–7.0)	6.4 (3.4–7.0)	0.01
Symptom domain	5.9 (1.8–7.0)	6.5 (3.5–7.0)	0.01
Emotional function domain	6.4 (2.1–7.0)	6.9 (2.9–7.0)	0.02
Total score	5.9 (2.3–7.0)	6.6 (3.7–7.0)	0.005
WHO-5 Wellbeing Index, %^a^	13 (7–24)	14 (5–25)	1

HR-QoL, health-related quality of life; WHO, World Health Organization

Data are presented as median values (range)

^a^ Only administered to children aged 12 years or over (27 and 47 subjects in the SA and NSPA groups, respectively)

No differences in eosinophils, total IgE and FE_NO_ levels were observed between the groups (Table 5). Children with SA had lower FEV_1_, FEV_1_/FVC and FEF_25–75_ z scores than their NSPA peers (*p* < 0.001 for each parameter). A normal FEV_1_ was found in 23 (56 %) SA and 69 (88 %) NSPA patients (*p* < 0.001).

**Table 5:** Laboratory results of children with severe asthma (SA) and non-severe persistent asthma (NSPA)

	SA	NSPA	*p*
	(*n* = 41)	(*n* = 78)	
Eosinophil count, 10^6^ · L^−1^	420 (10–1340)	485 (3–2420)	1
Serum total IgE levels, kUA/L	506 (27–4100)	541 (39–15850)	0.9
FE_NO_, ppb	30 (2–196)	21 (2–156)	0.3
Spirometry data			
FVC, z score	−0.3 (−4.1–2.1)	0.2 (−3.4–2.9)	0.08
FEV_1_, z score	−1.4 (−4.4–2.3)	−0.2 (−3.3–2.4)	<0.001
FEV_1_/FVC, z score	−1.7 (−3.7–2.9)	−0.1 (−2.9–3.0)	<0.001
FEF_25–75_, z score	−2.0 (−5.2–2.5)	−0.5 (−3.8–2.0)	<0.001

FE_NO_, fraction of exhaled nitric oxide; FVC, forced vital capacity; FEV_1_, forced expiratory volume at 1 s; FEF_25–75_, forced expiratory flow between 25 % and 75 % of vital capacity

Data are presented as median values (range)

## Discussion

The careful characterization of children with SA is considered an important step towards improving the knowledge of this small but very challenging group of patients [2, 5]. This is one of the few studies describing the distinguishing features of SA in children and adolescents in Europe. Hospital admissions for asthma, emergency-department visits during the past year, symptoms triggered by physical activity, lower spirometric values, and worse HR-QoL, but not well-being index, were differentiating features of SA *versus* NSPA. Current signs and symptoms possibly associated with atopy (i.e. rhinoconjunctivitis and eczema), eosinophil count, total IgE levels, and FE_NO_ values were similar in children with SA and NSPA, while lifetime sensitization to food allergens was an independent factor associated with SA. Home environment was similar in both groups, even though children with SA had a borderline higher prevalence of smoking parents.

SA in children is a challenging disorder with significant public health implications [1, 2, 19]. Unsurprisingly, in this study the occurrence of hospital admissions and emergency-department visits for asthma was a discriminating feature of SA *versus* NSPA. Nevertheless, 20 % of patients with SA had never been hospitalized, 71 % and 54 % were not hospitalized or admitted to the emergency department during the year preceding the study, and none had been admitted to intensive care units during the previous year. These findings are consistent with an earlier report from Sweden [9], where criteria for patient inclusion were similar to ours, while children enrolled in 5 specialized USA centers participating in the Severe Asthma Research Program [7] and those recruited in 12 specialized French centers at the beginning of Omalizumab treatment [20] had more severe exacerbations or at least required a larger utilization of health care services. Furthermore, most of our patients with SA regularly play a sport and are as physically active as their peers with NSPA.

Previous studies have highlighted an association between increasing asthma severity in children and both reduced HR-QoL and parents’ work attendance [10, 21, 22]. Asthma symptoms not only affect children physically, but also impair them and their families socially and emotionally [10, 21, 22]. In line with previous observations, this study demonstrates that poor HR-QoL strongly discriminates SA from NSPA. Interestingly, in our study girls with SA reported a significantly better HR-QoL than boys. Such gender distinction could reflect different psychological responses to limitations imposed by asthma (e.g. on physical activity) rather than actual differences in the disease itself. Moreover, our patients with SA had similar Wellbeing Indexes to peers with NSPA, with no gender differences. Of note, both groups had a median index very close to the poor wellbeing threshold (set at 13), the cut-off value below which it is recommended to test the patient for depression [14]. This finding has never been reported in children with SA and is consistent with the observation that the prevalence of anxiety and depressive disorders is significant among asthmatic patients, particularly adolescents [19, 23]. A more in-depth analysis of psychological aspects in children and adolescents with SA would be worthy also for identifying specific interventions that could help reduce asthma morbidity.

According to some [8, 10] but not all [4] previous studies, most of our SA patients were sensitized to aeroallergens, with no prevalence difference from the NSPA group. Nevertheless, we highlight the novel finding that lifetime sensitization to food allergens was more frequent in SA than in NSPA. Although children with asthma show a strikingly high prevalence of food sensitization [24, 25], the majority have no clinical food allergies [25], which is consistent with the presence of current food allergies in only a third of our children sensitized to food. It is well known that children with food sensitization have increased asthma morbidity, with a higher hospitalization frequency and greater need for steroid medications [26], and even if they have developed tolerance to food allergens by school-age, previous sensitization still represents a risk factor for later asthma development [27, 28]. Our results confirm and extend these findings by demonstrating that lifetime sensitization to food allergens is an independent risk factor for SA.

Reduction in FEV_1_ is often used to define childhood asthma severity in treatment guidelines [1] and clinical studies [7, 9, 29]. Interestingly, while our SA patients showed worse spirometric measures than their NSPA peers, more than half had a normal FEV_1_, indicating that a reduction in FEV_1_ is an insensitive measure of SA. Indeed, contrary to adults, spirometry may be a poor predictor of asthma severity in children, and previous studies examining the relationship between FEV_1_ and the childhood asthma severity level have demonstrated very weak correlations between lung function and asthmatic symptoms [19, 29, 30]. FE_NO_ levels were similar in both groups, in line with some previous studies showing no significant increase in FE_NO_ values in SA children [9, 21, 29]. As all our subjects were on regular steroids maintenance therapy, the lack of any difference in FE_NO_ between SA and NSPA may be explained by the anti-inflammatory effects of the treatment.

Unlike previous studies [8–10], a number of comorbidities (i.e. rhinoconjunctivitis, obesity, and symptoms of sinusitis and of gastroesophageal reflux), family characteristics (namely, parental asthma and education), and environmental exposures (i.e. dampness at home and exposure to heavy traffic) were similar in SA *versus* NSPA patients. Exposure to smoking at home was indeed more prevalent in patients with SA, although the difference did not reach statistical significance possibly because of the low sample size.

We used a national online web-based system to collect a large number of personal, family and environmental data from children with SA and NSPA enrolled in various Italian centers. The main strengths of this system are that it allows for collecting huge amounts of longitudinal data, and ideally enables inclusion of foreign patients for larger international studies. Another strength of our study is that, unlike previous reports [6–8, 10], we included only children with refractory asthma or in whom treatment of comorbidities has been addressed as per the ERS/ATS Guidelines definition [2].

This study has limitations. Firstly, the study was not designed to assess the prevalence of SA in Italy, which would have been difficult to achieve considering the extension of the country. Secondly, the number of patients recruited was small and this prevented us from drawing definite conclusions on the lack of difference in a few variables between patients with SA and NSPA. However, our sample size is similar to that of previous European studies [8–10]. Finally, exposure to smoke and adherence to prescribed medication were assessed via self-reporting. Nonetheless, the lack of any adherence differences between SA and NSPA suggests that this shortcoming did not have much impact on our findings.

## Conclusions

This study shows that, compared to children and adolescents with NSPA, discriminating features of SA include lifetime sensitization to food allergens, worse airway obstruction, and increased use of health-care resources. Apart from a greater exposure to parental smoking, environmental, family and personal characteristics as well as comorbidities of our SA patients are not different from those of their peers with NSPA. Finally, SA patients have worse HR-QoL than NSPA peers, and the perception of wellbeing is borderline in both groups, suggesting that the psychological aspects in these patients deserve further investigation.

## Additional files

Additional file 1:
**Criteria for exclusion of children with severe asthma and non-severe persistent asthma.** (PDF 11 kb) Additional file 2:
**Criteria for inclusion of children with severe asthma and non-severe persistent asthma.** (PDF 25 kb)

## Abbreviations

ATS: American thoracic society
BMI: body mass index
ERS: European respiratory society
FE_NO_: fraction of exhaled nitric oxide
HR-QoL: health-related quality of life
NSPA: non-severe persistent asthma
SA: severe asthma
