# Supplementary material for: Severe asthma features in children: a case–control online survey
Source: Ital J Pediatr. 2016 Jan 22;42:9. doi: 10.1186/s13052-016-0217-z (PMC4722711; doi:10.1186/s13052-016-0217-z)
Supplement: Additional file 1: — Criteria for exclusion of children with severe asthma and non-severe persistent asthma. (PDF 11 kb) [file 13052_2016_217_MOESM1_ESM.pdf]

E-table 1. Criteria for exclusion of children with severe asthma and non-severe persistent asthma.

Dysfunctional breathing/vocal-cord dysfunctions
Prematurity and related lung diseases
Cystic fibrosis
Congenital or acquired immune deficiencies
Primary ciliary dyskinesia
Bronchiolitis obliterans
Bronchiectasis
Pulmonary fibrosis and interstitial lung disease
Foreign body inhalation
Tracheo-bronchomalacia
Congenital malformations including vascular ring
Tracheal stenosis or membrane
Pulmonary cystic or mass lesions
Malignancies
Mediastinal mass/enlarged lymph nodes
Heart disease
Serious neurological diseases
Recurrent (micro)aspiration/swallowing dysfunctions
Connective tissue diseases
Previous major lung surgery
Central airway obstruction/compression
