# Supplementary material for: Severe asthma features in children: a case–control online survey
Source: Ital J Pediatr. 2016 Jan 22;42:9. doi: 10.1186/s13052-016-0217-z (PMC4722711; doi:10.1186/s13052-016-0217-z)
Supplement: Additional file 2: — Criteria for inclusion of children with severe asthma and non-severe persistent asthma. (PDF 25 kb) [file 13052_2016_217_MOESM2_ESM.pdf]

E-table 2. Criteria for inclusion of children with severe asthma and non-severe persistent asthma.

Severe asthma	Non-severe persistent asthma
Asthma which requires treatment with high doses of ICS *plus* a second controller (long-acting inhaled bronchodilator, leukotriene modifier or theophylline) for ≥50% of the previous year or systemic corticosteroids for ≥50% of the previous year to prevent it from becoming 'uncontrolled', or remaining 'uncontrolled' despite treatment.	Asthma which requires treatment with daily medium doses of ICS for the previous year to prevent it from becoming 'uncontrolled'. Use of other controllers is acceptable.
Threshold daily dose of ICS in µg:<br>- Beclomethasone dipropionate, 800 (DPI or CFC MDI) or 320 (HFA MDI) for children aged 6-12 yrs and 1000 (DPI or CFC MDI) or 500 (HFA MDI) for children aged >12 yrs;<br>- Budesonide, 800 both for children aged 6-12 yrs and children aged >12 yrs;<br>- Fluticasone propionate, 500 both for children aged 6-12 yrs and children aged >12 yrs;<br>- Mometasone furoate, 500 for children aged 6-12 yrs and 800 for children aged >12 yrs.	Threshold daily dose of ICS in µg:<br>- Beclomethasone dipropionate, 200;<br>- Budesonide, 200;<br>- Fluticasone propionate, 200;<br>- Mometasone furoate, 200.
At least one of the following during the previous year:<br>- at least 2 exacerbations of asthmatic symptoms each requiring bursts of systemic corticosteroids for >3 days;<br>- persistent symptoms or symptoms limiting daily activities (including sport or leisure activities) more than twice a week for at least 3 consecutive months;<br>- nocturnal symptoms more than twice a week for at least 3 consecutive months;<br>- persistent airflow obstruction (FEV_1 <2 standard deviations for age) despite administration of oral steroids and bronchodilators for at least 2 weeks.	All of the following during the previous year:<br>- less than 2 exacerbations of symptoms each requiring bursts of systemic corticosteroids for >3 days;<br>- occasional symptoms related to strenuous exercise only, otherwise no symptoms;<br>- no or only sporadic nocturnal symptoms.

ICS: inhaled corticosteroids; DPI: dry-powder inhaler; CFC: chlorofluorocarbon; MDI: metered-dose inhaler; HFA: hydrofluoroalkanes.
